# Integrated analyses reveal the diagnostic and predictive values of COL5A2 and association with immune environment in Crohn’s disease

**DOI:** 10.1038/s41435-024-00276-5

**Published:** 2024-05-24

**Authors:** Tingting Zhong, Xiaoqing Cheng, Qianru Gu, Guoxiang Fu, Yihong Wang, Yujie Jiang, Jiaqi Xu, Zhinong Jiang

**Affiliations:** Department of Pathology, Sir Run Run Shaw Hospital, Zhejiang University School of Medicine, Hangzhou, China

**Keywords:** Genetic markers, Translational immunology

## Abstract

The pathogenesis of Crohn’s disease (CD) involves abnormal immune cell infiltration and dysregulated immune response. Therefore, thorough research on immune cell abnormalities in CD is crucial for improved treatment of this disease. Single-cell RNA sequencing (scRNA-seq) and bulk RNA-seq data of CD were obtained from the Gene Expression Omnibus (GEO) database. Cell-type identification by estimating relative subsets of RNA transcripts (CIBERSORT), weighted gene co-expression network analysis (WGCNA), protein–protein interaction (PPI) networks evaluated the proportion of immune infiltrating cells, constructed co-expression network and identified key genes, respectively. Based on the dataset (GSE134809), 15 cell clusters were defined and labeled as different cell types. Among the 11 modules, the yellow module had the closest relationship with plasma cells (cluster 5). Confirmed using RNA sequencing and IHC assay, the expression of COL5A2 in CD samples was higher than that in control samples. Furthermore, the COL5A2 protein expression remarkably decreased in the group of patients who responded to anti–tumor necrosis factor (TNF) treatments, compared to the non-response group. The comprehensive analyses described here provided novel insight into the landscape of CD-associated immune environment. In addition, COL5A2 were identified as potential diagnostic indicators for CD, as well as promising predictive markers for CD patients.

## Introduction

Crohn’s disease (CD) is a subtype of inflammatory bowel disease (IBD), characterized by recurrent bouts of uncontrolled inflammation [1]. Its incidence and prevalence are rising globally and becoming a greater public health burden [2, 3]. Aminosalicylates, corticosteroids, immunomodulators, and biological agents are the current advised medical therapy for CD. Cytokine tumor necrosis factor (TNF) blockage has been a mainstay for over two decades after its first use in patients with CD [4–7]. TNF antagonists have primary non-response rates between 10% and 40%, and secondary loss of response can occur in up to 46% of patients within the first 12 months [4, 8–10]. Therefore, biomarkers in the early diagnosis of CD are urgently needed to predict therapy response prior to the first treatment to avoid unnecessary expenses and complications.

Although the exact cause of CD remains unclear, it is considered to have a complex etiology that involves an interplay of environmental factors, genetic susceptibility, disruption of the epithelial barrier, and an uncontrolled immune response to intestinal triggers [11–13]. Therefore, exploring the function of immune environment and related cytokines is crucial in developing effective treatment strategies for CD. Single-cell RNA sequencing (scRNA-seq) provides valuable information for single-cell characterization and enables the investigation of the hidden heterogeneity in cell populations by using optimized next-generation sequencing technologies [14, 15]. Recent studies have utilized scRNA-seq to explore the characteristics of cell subsets and their interactions in a range of malignancies [16–18]. Another recent study employed scRNA-seq to identify the GIMATS module, a unique cellular module linked to CD patients’ failure to achieve sustained remission upon anti-TNF therapy [11]. However, due to the relative high cost of scRNA-seq, the researchers can only obtain the gene expression profiles of single cells from a very small number of samples.

In the current investigation, CD inflamed tissues and uninflamed tissues were analyzed using scRNA-seq (GSE134809) dataset from GEO database to identify distinct cell subsets. Co-expression networks and critical modules in CD were screened using the weighted gene co-expression network analysis (WGCNA), based on the expression profile of the GSE101794 dataset from CD and normal samples. Furthermore, crucial genes related to immune cell subsets were explored based on the integration of bulk RNA-seq and scRNA-seq data. The diagnostic and predictive role of COL5A2 and COL4A2 were evaluated by performing mRNA sequencing and immunohistochemistry (IHC) of fresh-frozen samples and paraffin-embedded specimens, respectively. These genes show great potential as diagnostic and predictive biomarkers, as well as therapeutic targets for CD patients.

## Materials and Methods

### Data download

The CD datasets were downloaded from the GEO database, which included GSE134809 [11] (11 paired ileum tissues from the inflamed and uninflamed regions), GSE101794 [19] (the ileum biopsies from 254 patients with CD and 50 non-IBD controls), GSE93624 [20] (the ileum tissues from 210 patients with CD and 35 non-IBD controls), GSE10616 [21] (the colon biopsies from 32 patients with CD and 16 normal controls), GSE75214 [22] (the ileum tissues from 67 patients with CD and 11 healthy controls, and the colon tissues from 8 patients with CD and 11 healthy controls), and GSE16879 [23] (73 CD tissues from intestinal mucosal biopsies before and after infliximab treatment).

### Single-cell RNA-seq analysis

The raw data contained a total of 101,375 cells. The proportions of mitochondria and rRNA were counted using the PercentageFeatureSet function. The filtering criteria included > 500 genes expressed by each cell and < 35% of mitochondria. Further, data normalization was applied using the LogNormalization method. After principal component analysis, unsupervised clusters and unbiased visualization of cell populations were performed on a two-dimensional map using uniform manifold approximation and projection (UMAP), a nonlinear dimensionality reduction method. A total of 15 subgroups were then screened for marker genes using the FindAllMarkers function with Minpct = 0.35 (expression ratio of the least differential gene) and logfc = 0.5 (fold difference). The marker genes were screened with corrected *p* < 0.05. Different cell types were identified using the SingleR package [24]. Moreover, pathway analysis was performed on 15 subsets using the ReactomeGSA package, a new tool for multi-omics and multi-species pathway analysis with the analyse_sc_clusters function [25].

### Cell-type identification by estimating relative subsets of RNA transcripts (CIBERSORT) and weighted gene co-expression network analysis (WGCNA)

CIBERSORT is a computational method for estimating the relative proportions of different cell types from mixed cell populations based on gene expression data. The differences among 12 cells in distinct groups in the GSE101794 data were estimated using the CIBERSORT analysis. In addition, the score of each sample relative to each cluster was evaluated using the CIBERSORT algorithm.

WGCNA is a computational approach used for identifying modules of highly correlated genes and exploring their relationships in biological systems through the construction of gene co-expression networks. The gene modules most relevant to a subset were screened using the WGCNA analysis on the similarity score. For crucial genes, genes with module membership (MM) > 0.7 and gene significance (GS) > 0.4 were selected.

### Protein-protein interaction (PPI) network analysis

PPI refers to the physical interactions between proteins, playing a crucial role in various biological processes and cellular functions. A PPI network for hub genes was built using the STRING online database and visualized using the Cytoscape (version 3.9.1) software, with the PPI interaction score of > 0.7 as statistically significant. Besides, a Cytoscape plug-in, molecular complex detection (MCODE), identified key clusters of the PPI network.

### Human sample collection

The collection of human samples was conducted with the approval of the human ethics committee of Sir Run Run Shaw Hospital, affiliated with Zhejiang University (2022-0293). All patients signed informed consent forms provided by Sir Run Run Shaw Hospital. CD ileum tissues from inflamed and uninflamed areas were collected during the first surgical resection from January 2019 to December 2020. Total RNA was isolated, and gene expression profiles were determined using RNA-Seq as previously described [26].

### Gene functional enrichment analysis

The “clusterProfiler” R package was used to perform the Gene Ontology (GO) enrichment analysis and Kyoto Encyclopedia of Genes and Genomes (KEGG) pathway. GO analysis was classified into three classes, including biological processes (BP), cellular components (CC), and molecular functions (MF).

### Immunohistochemistry (IHC) assay

Formalin-fixed and paraffin-embedded tissues were collected from 25 pre-treatment CD samples and 16 healthy control samples at the Sir Run Run Shaw Hospital from January 2019 to December 2021. The IHC assay was performed using an envision detection system (DAKO, Denmark) according to the manufacturer’s instructions [27]. The COL5A2 antibody (TA313657, 1:200) was purchased from OriGene, and PBS was used as a negative control. The proportion of positively stained cells was graded into four categories: 0, < 5%; 1, 6%–33%; 2, 34%–66%; and 3, > 67%. The immunostaining intensity was also graded into four categories: 0, negative; 1, weak; 2, moderate; and 3, strong. The cells in the ulcers were not included in the scoring. The IHC scores were obtained by multiplying the percentage and intensity scores. The scores for all the slides were evaluated independently by two pathologists to avoid observer bias.

### LncRNA–miRNA–mRNA network construction

The miRnada, miRDB and TargetScan databases were used to predict the corresponding miRNAs of COL5A2. The overlapping results of the three miRNA-mRNA databases were considered as miRNA-mRNA relationships. The spongeScan database was used to predict the upstream lncRNAs of the selected miRNA-mRNA relationships. The final ceRNA network was visually analyzed by the Cytoscape software.

### Statistical analysis

All analyses were conducted using R software 4.1.1 and GraphPad Prism v. 8.01 (GraphPad Software, CA, USA). The receiver operating characteristic (ROC) curve was used to analyze the diagnostic and predictive value of gene expression in CD patients. Furthermore, the values between distinct groups were compared using the Mann–Whitney U-test or Student’s t-test. A *p*-value < 0.05 was considered statistically significant.

## Results

### Single‑cell RNA‑seq profiling and clustering

**Fig. 1:**
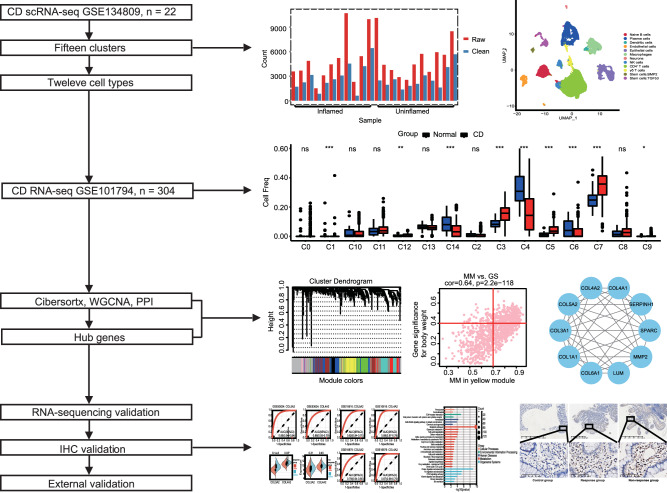
Flow chart of this study. Schematic overview of all steps and results.

**Fig. 2:**
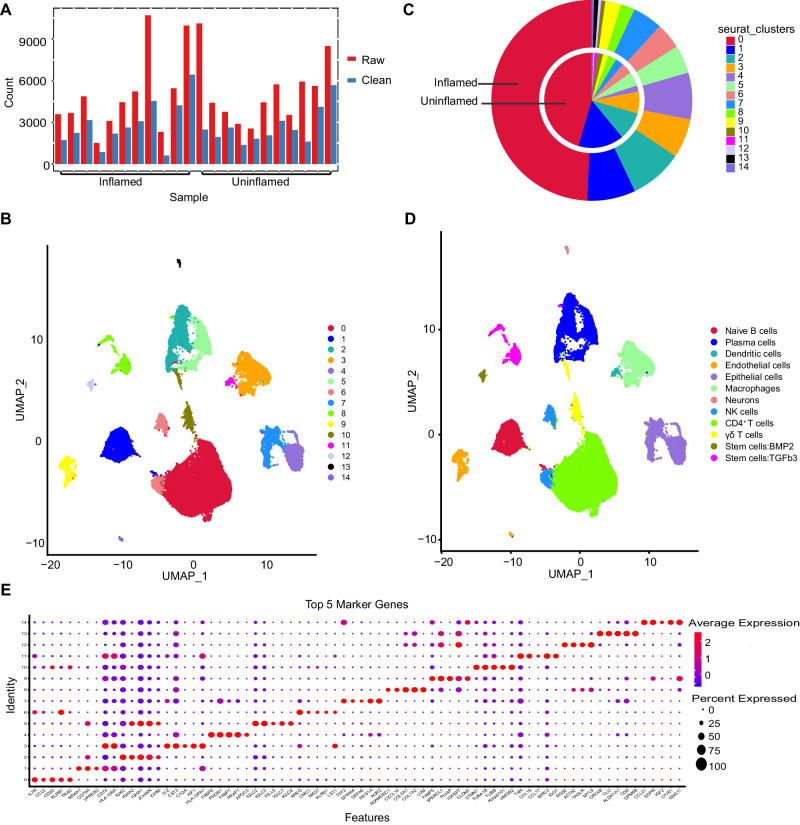
Analysis of single-cell RNA sequencing of 11 paired CD tissues. **A** Number of cells before and after filtration. **B** UMAP of the 15 cell clusters. **C** Proportions of each cluster in the inflamed and uninflamed groups. **D** Cells clustered into 12 types. **E** Top 5 marker genes expressed in each cell type.

The overall flow scheme of the study is shown in Fig. 1. After pretreating scRNA-seq data (Fig. S1), 57,748 cell samples were screened from 11 pair CD tissues (Fig. 2A). The scRNA-seq data were visualized using the UMAP technique, and the cells were successfully classified into 15 clusters (Fig. 2B). The proportions of the subsets in CD and uninflamed groups are shown in Fig. 2C. All clusters were annotated with the SingleR R software package to acknowledge cell types (Fig. 2D). CD4^+^ T cells, γδT cells, naïve B cells, plasma cells, epithelial cells, endothelial cells, dendritic cells, monocytes, natural killer (NK) cells, neurons, and tissue stem cells were characterized. In addition, significantly expressed marker genes were identified between groups with a threshold of logFC > 0.5, Minpct = 0.35, and adjpval < 0.05. The first five significantly different markers for each cluster are shown in Fig. 2E. The functional enrichment of all subgroups was analyzed by ReactomeGSA R package, and the difference between the maximum and minimum values was calculated for each pathway score (Supplementary Table 1).

### Identification of key genes using CIBERSORT, WGCNA, and PPI network

**Fig. 3:**
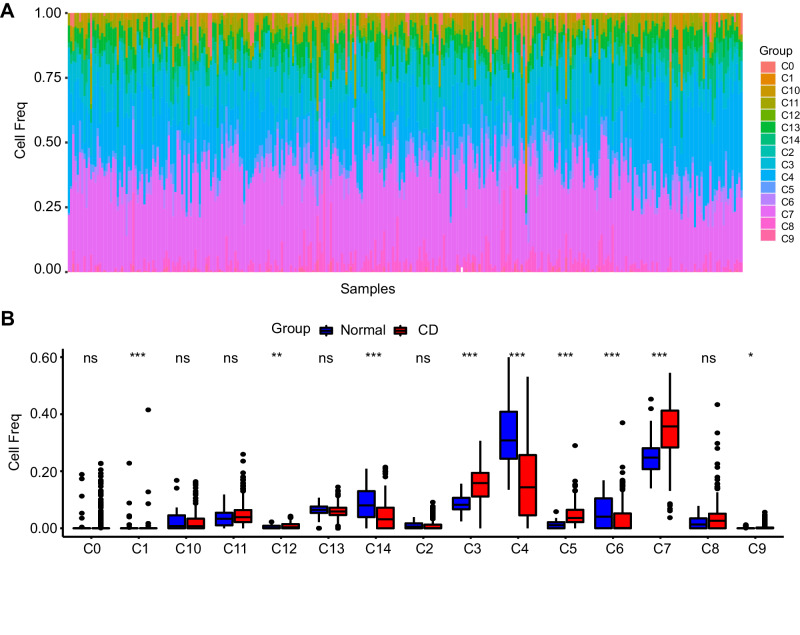
Fractions of immune cells. **A** Heatmap of the proportion of 15 clusters built on the GSE101794 database. **B** Abundances in CD and normal tissues. *P < 0.05; **P < 0.01; and ***P < 0.001.

On the basis of the results of scRNA-seq data, the proportions of 15 clusters from the GSE101794 database was predicted using CIBERSORT (Fig. 3A). In a comparison between 254 CD samples and 50 normal samples, distinct variations in the percentages of nine clusters were observed, including C1, C3, C4, C5, C6, C7, C9, C12, and C14, corresponding to seven cell types: naive B cells, macrophages, epithelial cells, plasma cells, NK cells, endothelial cells and stem cells:BMP2 (Fig. 3B).

**Fig. 4:**
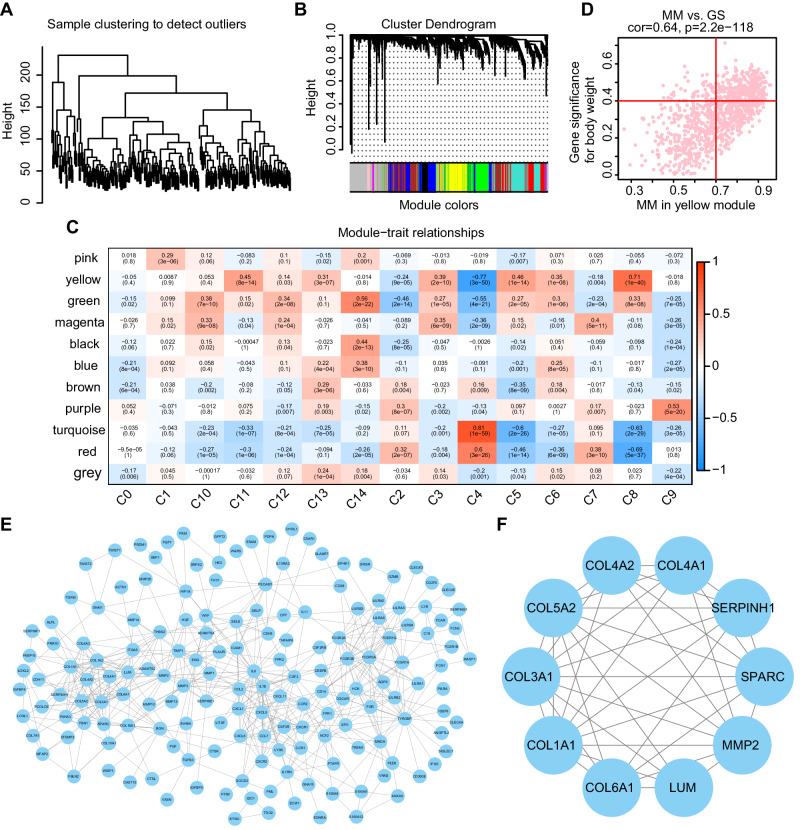
Hub gene selection. **A** Sample clustering to detect outliers. **B** Gene clustering dendrogram based on different metrics. The color lines below the tree dendrogram indicate the module colors. **C** Correlation analysis between each module and cell subgroup. **D** A scatter plot of the correlation between GS for body weight and MM in the yellow module, with *p* = 2.2e − 118 and correlation = 0.64. Red dots identify as hub genes. **E** PPI network for the hub genes, with each round node representing a gene. **F** Ten key genes were identified in cluster 1 using the MCODE plugin.

The correlation between distinct cell subsets and gene expression patterns in CD was explored using the WGCNA method based on the GSE101794 data. The hierarchical clustering analysis of every sample was depicted in Fig. 4A. Applying the WGCNA approach, a threshold of 12 was selected to induce a scale-free distribution in the sample, and a scale-free network was constructed to identify gene characteristics. Then 11 modules were generated without grey module, which did not aggregate with other modules (Fig. 4B). Moreover, the correlation between every module and the proportions of cell subsets were analyzed (Fig. 4C). CD was closely associated with the yellow module, which was significantly related with the plasma cells score (*r* = 0.46, *p* = 1e − 14). Next, the GS and MM values were calculated in all modules, and the hub genes were obtained from the yellow module (GS > 0.4, MM > 0.7) (Fig. 4D).

Furthermore, the interactions among the hub genes were investigated, and the PPI network was constructed, containing 165 nodes and 527 edges (Fig. 4E). All clusters in the network were found using the MCODE, and 8 clusters were counted according to k-core = 2. The cluster 1 had the highest score (8.444) among them, including 10 nodes and 38 edges (Fig. 4F). These key genes, including COL1A1, MMP2, COL5A2, SERPINH1, COL4A2, COL3A1, SPARC, COL4A1, COL6A1, and LUM, were matrisome-related genes [28].

### Diagnostic values of COL5A2 and COL4A2 in CD

**Fig. 5:**
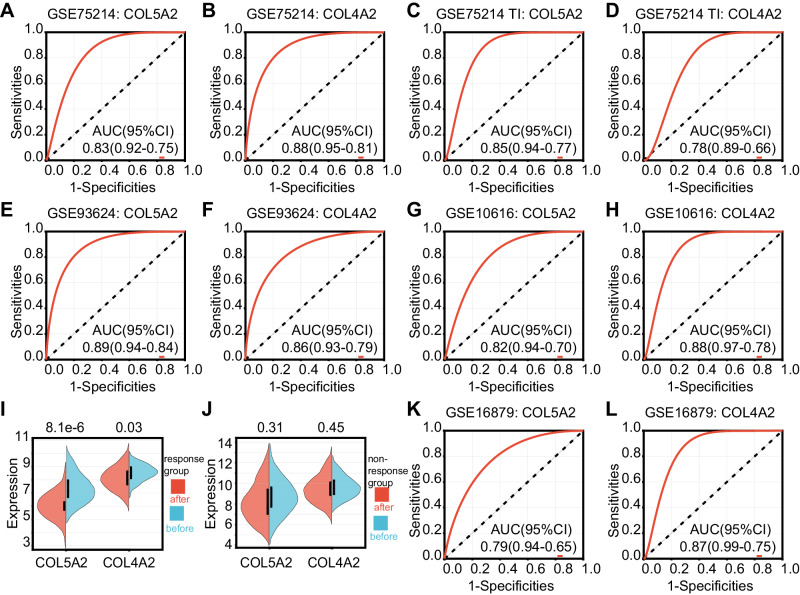
Diagnostic and predictive values of COL5A2 and COL4A2 in CD. **A**, **B** Diagnostic value in CD patients using all mucosal samples (GSE75214 dataset). **C**, **D** Diagnostic value in CD patients using terminal ileum samples (GSE75214 dataset). **E**, **F** Diagnostic value in CD patients (GSE93624 dataset). **G**, **H** Diagnostic value in CD patients (GSE10616 dataset). **I**, **J** Expression in CD samples that underwent infliximab therapy in response group and non-response group. **K**, **L** Predictive values in the treatment of CD.

Among the identified genes, both COL5A2 and COL4A2 showed the higher degrees of interaction; therefore, we considered these as potentially crucial genes in CD pathogenesis. The diagnostic values of COL5A2 and COL4A2 in patients with CD was investigated using the GSE75214 dataset to conduct ROC analysis as the training dataset. The area under the curve (AUC) values of COL5A2 and COL4A2 were 0.83 and 0.88, respectively, when all mucosal samples were conducted (Fig. 5A, B). However, the AUC values of the two genes were 0.85 and 0.78, respectively, when only terminal ileum tissues were employed (Fig. 5C, D). In addition, the AUC values of the two genes were above 0.9, respectively, when the colon tissues were analyzed. The results are shown in Fig. S2.

The results were validated using another two datasets, including GSE93624 and GSE10616. The diagnostic values of COL5A2 and COL4A2 were similar to those from the GSE75214 dataset, with 0.89 and 0.86 in the GSE93624 dataset (Fig. 5E, F) and 0.82 and 0.88 in the GSE10616 dataset, respectively (Fig. 5G, H). These findings indicated that the two key genes had good diagnostic performance in patients with CD, whether using ileum or colonic mucosal tissues.

### Predictive values of COL5A2 and COL4A2 in the treatment of CD

The GSE16879 dataset offered the concrete data before and after the first infliximab treatment as well as response or non-response data on treatment. The relative expressions of COL5A2 and COL4A2 were significantly downregulated after infliximab therapy in response group (Fig. 5I). Similarly, the relative expressions of COL5A2 and COL4A2 showed a downward trend in non-response group (Fig. 5J).

The AUC values for COL5A2 and COL4A2 were 0.79 and 0.87, respectively, when predicting the effect of infliximab treatment in CD (Fig. 5K, L). Overall, the results suggested that these two genes were promising markers for monitoring and predicting the efficacy of infliximab in CD.

### Our validation cohort using RNA sequencing

**Fig. 6:**
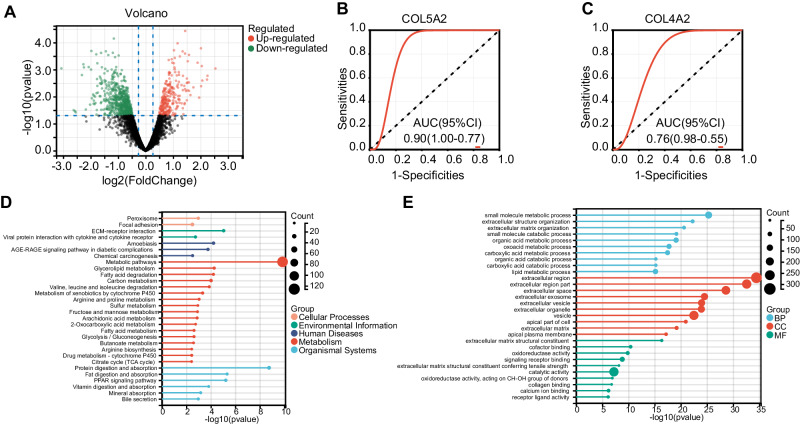
DEGs in our validation cohort. **A** Volcano plot of DEGs. **B**, **C** Diagnostic value of COL5A2 and COL4A2 in CD. Bar chart of (**D**) KEGG and (**E**) GO functional enrichment analysis. BP biological process, CC cellular component, MF molecular function.

A total of 12 mucosal tissues of patients with CD were obtained from the inflamed and uninflamed areas, and the gene expression profiles were analyzed with thresholds of |Log2 FC | > 0.5 and *p*-value < 0.05, using the “Limma” R package. The demographics and clinical characteristics are listed in Table 1. As shown in Fig. 6A, 272 upregulated and 614 downregulated genes were identified in the CD group. The AUC values of COL5A2 and COL4A2 in patients with CD were 0.90 and 0.76, respectively (Fig. 6B, C).

**Table 1:** Demographic and clinical characteristics of CD patients.

Characteristics	Surgical samples *N* = 12 (%)	Biopsy specimens *N* = 25 (%)
Gender (male)	5 (41.7)	17 (68.0)
Age at diagnosis (years), mean ± SD	39. 7 ± 17.4	25.4 ± 8.9
Sampling site		
Ileum	8 (66.7)	10 (40.0)
Ileocecal	2 (16.7)	3 (12.0)
colon	2 (16.7)	12 (48.0)
Montreal classification		
A1 ( ≤ 16)	2 (16.7)	4 (16.0)
A2 (17–40)	4 (33.3)	4 (16.0)
A3 ( > 40)	6 (66.7)	17 (68.0)
Location		
L1 (ileal)	2 (16.7)	4 (16.0)
L2 (colonic)	1 (8.3)	3 (12.0)
L3 (ileocolonic)	9 (75.0)	18 (72.0)
L4 (upper gastrointestinal)	2 (16.7)	10 (40.0)
Behavior		
B1 (inflammatory/failure of medical therapy)	1 (8.3)	16 (64.0)
B2 (stricturing)	8 (66.7)	4 (16.0)
B3 (penetrating)	10 (83.3)	5 (20.0)
Perianal disease	6 (66.7)	17 (68.0)

KEGG enrichment analysis showed that DEGs were primarily enriched in the top five pathways, including metabolic pathways, fat digestion and absorption, protein digestion and absorption, PPAR signaling pathway, and ECM-receptor interaction (Fig. 6D). GO enrichment analysis demonstrated that DEGs were correlated to extracellular-related biological processes, containing extracellular region (GO_CC), extracellular space (GO_CC), extracellular matrix organization (GO_BP), extracellular structure organization (GO_BP), and extracellular matrix structural constituent (GO_MF) (Fig. 6E). COL5A2 and COL4A2 were closely associated with the extracellular matrix, proving their importance in the pathogenesis of CD.

### Protein expression of COL5A2 in CD biopsy tissues

A total of 25 pre-treatment biopsies were obtained from patients with CD to avoid the influence of therapeutic drugs. The demographic and clinical data are described in Table 1. The patients were categorized according to infliximab response based on endoscopic and histological findings at 4–6 weeks after the first infliximab treatment and divided into two groups, including 14 cases in the response group and 11 cases in the non-response group.

**Fig. 7:**
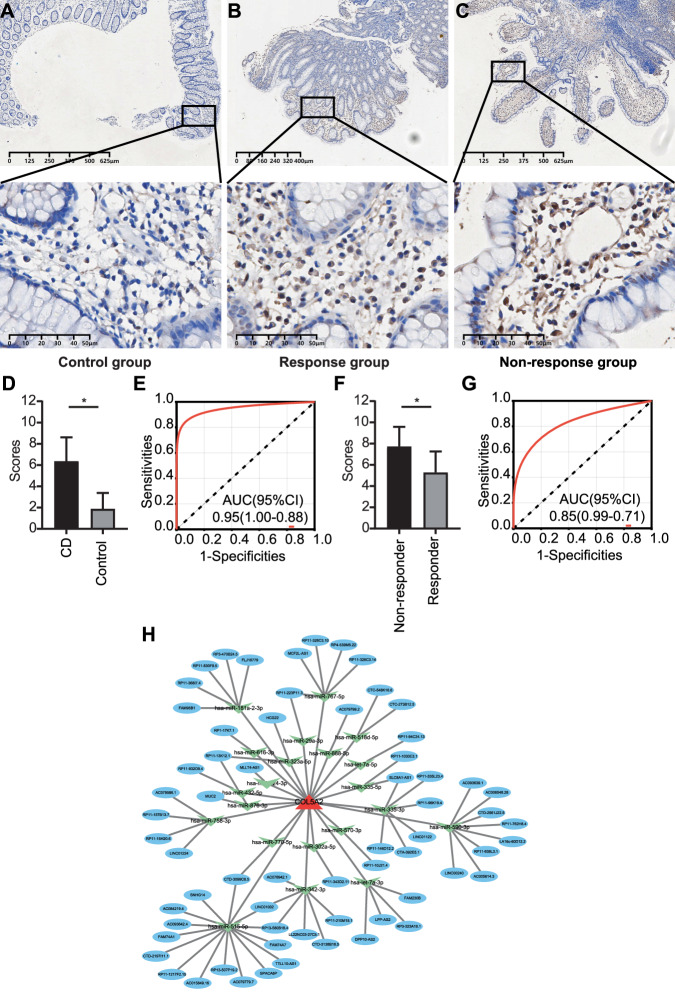
Analysis of COL5A2 expression using IHC staining. **A**–**C** Protein expression levels, and immunostaining was observed in the plasma cells (40× and 400× magnification). **D**, **E** Diagnostic value in patients with CD. **F**, **G** Predictive values of infliximab treatment in CD. IHC staining was assessed on a scale of 0–9. **H** LncRNA-miRNA-mRNA network analysis. Blue, green and red represent lncRNAs, miRNAs and COL5A2, respectively.

All samples were examined using IHC staining for COL5A2 expression, with the highest AUC value between the two genes from our sequencing data. It is generally believed that COL5A2, as a matrisome-related gene, is associated with fibrosis of CD and is highly expressed in the submucosal layer and muscular layer. However, most of the biopsies showed only mucosal layers. More importantly, we focused on the relationship between COL5A2 and immune cells in treatment-naive CD patients. The COL5A2 protein localized in different immune cells, primarily in plasma cells both in the cytosol and nucleoplasm, which was consistent with previous findings (Fig. 4C). Representative IHC images of COL5A2 protein expression in control, CD response, and CD non-response groups are shown in Fig. 7A–C. The COL5A2 expression was significantly increased in CD samples compared with the control samples (Fig. 7D), with the AUC value of 0.95 in the diagnosis of CD (Fig. 7E). Besides, COL5A2 protein expression was remarkably decreased in response group compared to non-response group (Fig. 7F), with the AUC value of 0.85 for predicting the efficacy of infliximab in CD (Fig. 7G). These findings suggested that COL5A2 was involved in the pathogenesis of CD. To further illuminate the potential mechanism of COL5A2 in CD, a lncRNA–miRNA–COL5A2 ceRNA regulatory network was built. The final ceRNA network contained 64 lncRNAs and 21 miRNAs, which reflected the potential RNA cross-talks in the process of CD (Fig. 7H).

## Discussion

Dysregulation of immune cell transport in the intestine is an important cause of CD pathogenesis [29]. Current treatment strategies typically focus on blocking the inflammatory response cascade and pro-inflammatory cytokines [30, 31]. In this study, we employed a variety of bioinformatics methods to analyze several datasets from the GEO database, identifying four distinct cell types that differ between CD and normal tissues: naive B cells, macrophages, plasma cells, and NK cells. These results are consistent with a previous study [32], confirming the crucial role of immune cells in CD pathogenesis and progression. Moreover, we unveiled COL5A2 as a novel biomarker with potential diagnostic value and predictive capacity for anti-TNF treatment responsiveness, elucidating its ties to immune cells and implicating it in CD pathophysiology.

Through WGCNA and PPI analysis, our investigations discovered that plasma cells and hub genes associated with plasma cells play a crucial role in the pathogenesis of CD. Studies on the role of plasma cell in CD and their underlying mechanism are extremely limited. In pathology, the inflammatory infiltration of plasma cells, particularly basal plasmacytosis, is the earliest diagnostic feature that distinguishes CD from non-IBD [33]. Brandtzaeg et al. reported that CD involves B cell destruction, with elevated plasma cell levels in the lamina propria and changes in antibody subclasses [34]. Maria et al. pointed out that in CD and ulcerative colitis, the gut mucosa had an increased presence of plasma cells that produced granzyme B, which indicated a potential role in the pathogenesis and tissue damage [35]. The localization of COL5A2, primarily in plasma cells both in the cytosol and nucleoplasma further supports their involvement in CD.

TNF family genes play important roles in immune regulation and inflammation [36]. Individual markers, such as Oncostatin M serum levels and triggering receptor expressed on myeloid cells-1 mRNA levels, are considered good predictors of anti-TNF response [37–41]. However, additional indicators are needed to improve the prediction accuracy. The present study identified COL5A2 as a valuable indicator for predicting the effect of anti-TNFα treatment. COL5A2 is a matrisome-related gene involved in multiple biological processes. Its mutations are associated with type I and II Ehlers–Danlos syndrome [42, 43]. Many studies have demonstrated that COL5A2 is differentially expressed in various tumors, such as gastric cancer, esophageal squamous cell carcinoma, non-small cell lung cancer (lung squamous carcinoma and lung adenocarcinoma), and colorectal cancer, and is associated with poor prognosis [44–48]. Besides, COL5A2 is a prognostic biomarker in gastric cancer, highlighting its correlation with immune infiltrates, such as CD4+ and CD8 + T cells, macrophages and plasmacytoid dendritic cells [49]. Recently, Meng et al. reported that COL5A2 was highly expressed in proliferative diabetic retinopathy and associated with M2 macrophages [50]. These results are consistent with our findings, which showed the association of COL5A2 and immune cells, especially plasma cells in patients with CD.

The results of KEGG and GO enrichment analysis revealed that COL5A2 was closely associated with the extracellular matrix. A study suggested that alterations in extracellular matrix homeostasis, caused by TNF-stimulated gene-6, support the formation of fistulas in CD through a process mediated by the epithelial-to-mesenchymal transition [51]. Another study pointed out that inhibiting fibroblast activation protein led to a reduction in collagen production, which could help restore extracellular matrix homeostasis and reduce fibrosis in fibrostenotic CD [52]. These studies collectively underscore the significance of extracellular matrix components in the pathology of Crohn’s Disease. Therefore, the relationship between COL5A2, extracellular matrix and effective treatments for CD patients can be further studied, especially in the context of strictures and fistulizing disease. A lncRNA–miRNA–COL5A2 ceRNA regulatory network may help further illuminate the potential mechanism of COL5A2 in CD.

In the present study, the immune environment and matrisome-related genes were explored in CD by analyzing the bulk RNA-seq and scRNA-seq data, with COL5A2 showing promising diagnostic and predictive values for patients with CD. Nonetheless, limitations of this study include the use of a small validation cohort and the lack of clinical information, such as disease phenotype, CD activity index score, and disease activity status, to accurately estimate the correlation between disease severity and immune cells. In addition, although the COL5A2 expression was verified in CD tissues using IHC, further validation is required to confirm its predictive power, especially due to the lack of tissues after anti-TNFα treatment.

## Conclusion

These comprehensive analyses provided valuable knowledge in the landscape of CD-associated immune environment and revealed matrisome-related COL5A2 and COL4A2 as new auxiliary diagnostic indicators for CD. In addition, these two genes show promise as predictive indicators in CD patients receiving anti-TNF therapy.

### Supplementary information

Supplementary material legends

Supplementary Figure 1

Supplementary Figure 2

Supplementary Table 1

## Data Availability

The datasets used in this research are available via public databases. The data and code that support the findings of this study are available from the corresponding author, upon reasonable request.
