# Supplementary material for: Integrated analyses reveal the diagnostic and predictive values of COL5A2 and association with immune environment in Crohn’s disease
Source: Genes Immun. 2024 May 24;25(3):209–18. doi: 10.1038/s41435-024-00276-5 (PMC11178494; doi:10.1038/s41435-024-00276-5)
Supplement: Supplementary file 1 — Supplementary material legends [file 41435_2024_276_MOESM1_ESM.docx]

**Integrated analyses reveal the diagnostic and predictive values of COL5A2 and association with immune environment in Crohn’s disease**

**Supplementary material legends**

Supplementary Figure 1 Quality-control filtration of each sequenced cell using violin plots to show the number of RNA features (nFeature_RNA), absolute UMI counts (nCount_RNA), the proportion of mitochondrial gene expression (percent.mt) and rRNA expression (percent.Ribo) in all genes. (A-D) before filtering; (E-H) after filtering.

Supplementary Figure 2 Diagnostic value in CD using colon tissues (GSE75214 dataset).

Supplementary Table 1 The functional enrichment of all subgroups.
