# Supplementary material for: Integrated analyses reveal the diagnostic and predictive values of COL5A2 and association with immune environment in Crohn’s disease
Source: Genes Immun. 2024 May 24;25(3):209–18. doi: 10.1038/s41435-024-00276-5 (PMC11178494; doi:10.1038/s41435-024-00276-5)
Supplement: Supplementary file 2 — Supplementary Figure 1 [file 41435_2024_276_MOESM2_ESM.pdf]

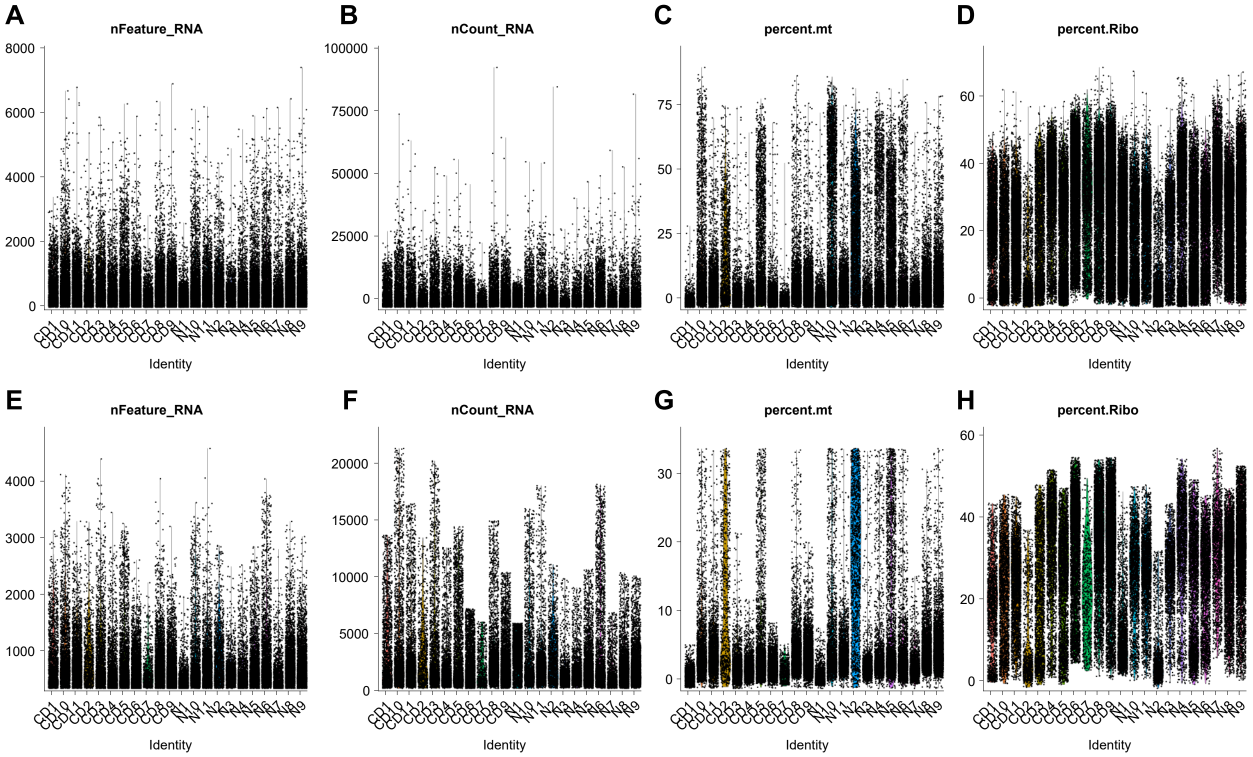

A
nFeature_RNA
8000
6000
4000
2000
0
Identity
B
nCount_RNA
100000
75000
50000
25000
0
Identity
C
percent.mt
75
50
25
0
Identity
D
percent.Ribo
60
40
20
0
Identity
E
nFeature_RNA
4000
3000
2000
1000
Identity
F
nCount_RNA
20000
15000
10000
5000
0
Identity
G
percent.mt
30
20
10
0
Identity
H
percent.Ribo
60
40
20
0
Identity
