# Supplementary figures and images for: Integrated analyses reveal the diagnostic and predictive values of COL5A2 and association with immune environment in Crohn’s disease
Source: Genes Immun. 2024 May 24;25(3):209–18. doi: 10.1038/s41435-024-00276-5 (PMC11178494; doi:10.1038/s41435-024-00276-5)

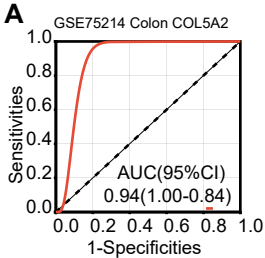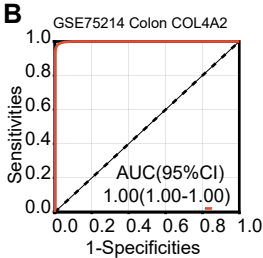

Supplement: Supplementary file 3 — Supplementary Figure 2 [file 41435_2024_276_MOESM3_ESM.pdf]
